# PROTOCOL: Child and adolescent mental health and psychosocial support interventions: An evidence and gap map of low‐ and middle‐income countries

**DOI:** 10.1002/cl2.1221

**Published:** 2022-02-16

**Authors:** Manasi Sharma, Camila Perera, Alessandra Ipince, Shivit Bakrania, Farhad Shokraneh, Priscilla Idele, David Anthony, Prerna Banati

**Affiliations:** ^1^ UNICEF Office of Research—Innocenti Florence Italy; ^2^ UNICEF West and Central Africa Regional Office Dakar Senegal

## Abstract

This is the protocol for a Campbell review. The objective of this evidence and gap map is to provide an overview of the existing evidence on the effectiveness of interventions aimed at promoting mental health and reducing or preventing mental health conditions among children and adolescents in low‐ and middle‐income countries.

## 1 BACKGROUND

### 1.1 The problem, condition or issue

All children have the right to opportunities to survive, grow and develop, within the context of physical, emotional and social well‐being, to achieve their full potential (UN, 2013). Mental health has been defined as ‘a state of well‐being in which the individual realises his or her own abilities, can cope with the normal stresses of life, can work productively and fruitfully, and is able to make a contribution to his or her community’ (WHO, 2004, p. 11). While this definition moves away from the conceptualisation of mental health as solely the absence of illness, most research and prevalence studies on children and adolescents focus on the mental health conditions that affect mood, thinking and behaviour.

Indeed, it is estimated that globally mental health disorders affect about one in seven children and adolescents aged 10–19 (UNICEF, 2021). The magnitude and nature of child and adolescent mental health conditions can be illustrated through several key figures. First, and despite significant variation, the worldwide pooled prevalence of mental health conditions among children ages 10‐19 is estimated at 27.5% for anxiety disorder and 12.7% for depression, which are often comorbid (UNICEF, 2021). Second, depression is among the leading causes of disability among young people while suicide is a leading cause of death among adolescents worldwide, the third among 15–19‐year‐old adolescent girls (UNICEF, 2021). Lastly, most mental health conditions originate early in life, with 50% arising before the age of 14% and 75% by the mid‐20s (Kessler et al., 2010; Solmi et al., 2021). Yet, despite its prevalence, the evidence on effective interventions addressing the mental health and psychosocial well‐being of children and adolescents has not been consistently gathered and mapped (Kieling et al., 2011).

Across the phases of life, experiences and environment present potential risks and opportunities for children and adolescents. Mounting evidence has shown that the first 1,000 days represent a unique opportunity for unparalleled cognitive growth and early stimulation which are central to healthy mental and emotional lives (Erskine et al., 2017; Klasen & Crombag, 2013; Patel et al., 2018). During the early years of a child's life, parents and caregivers are instrumental in shaping child development and behaviour through proper child nutrition, education and a nurturing and safe home environment. Middle childhood (5–9 years) are school‐going years that provide the context for early peer support and nurturing care through positive interactions as well as providing opportunities for building important life skills (Kieling et al., 2011).

Adolescence is a second window of opportunity, where critical period in brain development is taking place and where adolescents adopt and maintaining social and emotional habits and are engaged in identity formation. This period is characterised by a heightened salience of relationships with peers, which become key factors in shaping and directing young people's psychological development (Mitic et al., 2021). The onset of puberty at this stage brings unique mental health challenges compounded by physiological and emotional transitions, as well as sexual and risk‐taking behaviours. Late adolescence (15–19 years) is shaped by community and social and cultural expectations of acceptable behaviour, gender norms and roles, and the upper end of adolescence comes with pressure to secure employment and gain social and economic independence.

At the same time, evidence highlights that adolescence is a time when young people harness skills and traits that can foster resilience, or the learned capacity to deal more effectively with ongoing adversity (Lansford & Banati, 2018). Effective positive coping strategies and behaviours adopted and learned during these years can reap benefits into adulthood. In addition, adolescence offers an opportunity to overcome early adversities. Although most children can adequately recover and adapt using their own resources, childhood and adolescence are also vulnerable periods during which adverse experiences can negatively impact cognitive, emotional, and behavioural development. Exposure to adverse experiences during these and earlier years means that without care and support some children and adolescents may carry the mental and emotional costs for years to come (Haahr‐Pedersen et al., 2020). Indeed, research shows that in low‐resource settings, multiple, overlapping childhood adversities (e.g., violence, neglect, abuse, parental separation or substance use) are consistently associated with poor mental health (Jokinen et al., 2021; Kieling et al., 2011; Reed et al., 2012).

Despite the high burden and early onset, most mental health conditions remain unrecognised and untreated. A global systematic review of survey data in 2004 estimated that 70% of people aged 15 and older who were living with mental health conditions lacked access to adequate care (Kohn et al., 2004) and that this gap is higher in low‐ and middle‐income countries (LMICs), where most children and young people live (90%; Kieling et al., 2011). This estimate may have changed in the last decade, and it is important to assess the evidence and treatment gaps in LMICs in more recent years. Additionally, there is growing evidence of effective, affordable and culturally acceptable interventions from high‐income settings for preventing and treating mental health conditions that can be implemented in LMICs (Das et al., 2016). School‐based programmes can have significant positive effects on children and adolescents' well‐being, including reduced depression and anxiety and improved coping skills (Barry et al., 2013). Various promotion and prevention approaches have been successfully implemented and rolled‐out in health and community settings (Bradshaw et al., 2021; Das et al., 2016; Klasen & Crombag, 2013; Skeen et al., 2019). Parent and family‐focused interventions (i.e., psychoeducation, parent and family‐skills training, behavioural, psychosocial, and trauma‐focused cognitive behavioural therapy) may be beneficial to child and youth mental health and well‐being, as well as parenting behaviours and family functioning (Pedersen et al., 2019).

### 1.2 Why it is important to develop the evidence and gap map (EGM)

Investment in child and adolescent mental health prevention, promotion and care is essential but the evidence from this field is yet to be consistently systematically collected and mapped. An EGM would assist in generating a clearer picture of the available evidence on interventions to improve child and adolescent mental health in low‐resource settings, thereby informing future research, policy and practice.

Promoting, protecting and caring for children and young people's mental health plays a key role in achieving most of the 17 sustainable development goals (SDGs). More specifically, Goal 3 calls on Member States to ensure healthy lives and promote well‐being for all *at all ages*. More specifically, SDG target 3.4 is to reduce premature mortality from non‐communicable diseases and to promote mental health and well‐being. Effective mental health interventions could act as potential development goal accelerators (Sherr et al., 2020)—with provisions that lead to progress across domains of a child's life, and impacting multiple SDGs (Patel et al., 2018). However, mental health care is under‐prioritised and under‐funded, representing just up to 1% of national budgets in LMICs (Patel et al., 2018). In the context of meeting these global goals, identifying *what works* in the field of mental health and psychosocial support in low‐resource settings and mapping potential areas of investment for future research and programming gains urgency.

Early evidence from the COVID‐19 crisis indicates exacerbated mental health problems during the pandemic, with children and young people globally at risk of psychosocial distress, including anxiety, depression, and externalising behaviours, due to lockdowns, school closures and economic recession (UNICEF Office of Research—Innocenti, 2021). The need for greater investment in mental health interventions emerged as a key finding in a recent rapid review on the topic (UNICEF Office of Research—Innocenti, 2021), as well as the importance of targeting specific risk and protective factors across age groups in LMICs, where the research is limited. A lack of understanding of what works acts as a barrier to increasing investment in mental health care (McDaid et al., 2008). More work is needed to examine the state‐of‐the‐evidence of programmatic interventions on children's mental health in the first and second decades of life. As yet, no unified resource exists that provides an overview of the evidence of child and adolescent mental health interventions in these settings.

Against such a backdrop, UNICEF has renewed its commitment by setting a new goal to secure investment and action to support and protect the mental health of children and young people. At present, 90% of research on child and adolescent mental health has been conducted in high‐income countries and evidence from low‐resource settings is sparse (Kieling et al., 2011). An EGM would serve to identify where the evidence is abundant, but also where limited research and absolute gaps exist and increase the visibility of the available evidence (Saran et al., 2020). This resource will allow us to identify under‐researched areas, countries and population sub‐groups and inform the decisions of international donors, policymakers, practitioners and researchers as well as UNICEF's research priorities and programmatic actions. A visual representation of the evidence, in the form of a matrix of interventions, will allow practitioners, researchers, donors and policymakers to identify and focus on the areas of research that are more likely to inform their work.

#### 1.2.1 Existing EGMs and/or relevant systematic reviews

A brief desk‐based scoping was conducted to inform the objectives of this EGM, which identified several EGMs covering adjacent topics and themes, and systematic reviews that explore a subset of the interventions and outcomes being proposed for inclusion in this EGM.

The *Mega Map of Child Well‐Being Interventions in Low‐ and Middle‐Income Countries* (Saran et al., 2020) includes evidence on parenting interventions and associated mental health and psychosocial well‐being outcomes that are relevant to the scope of our EGM, but does not map the evidence on mental health promotion, prevention and care interventions. Another recent EGM on *Interventions for reducing violence against children in LMICs* (Pundir et al., 2020) provides a valuable snapshot of the impact of violence prevention interventions on child mental health outcomes. However, it does not purposefully include interventions to address child and adolescent mental health and the outcomes are not explored to the same level of detail as proposed here. In an EGM on *Adolescent Well‐Being in Low‐ and Middle‐Income Countries*, Bakrania et al. (2018) gathered psychosocial support interventions but excluded mental health outcomes. In the context of the COVID‐19 pandemic, a *Rapid Evidence and Gap Map of Virtual Care Solutions for Youth and Families to Mitigate the Impact of the COVID‐19 Pandemic on Pain, Mental Health, and Substance Use* identified a series of virtual psychological interventions for management of chronic pain among young people during the pandemic (Birnie et al., 2021). Lastly, an EGM on interventions for children and adolescents with disabilities is currently underway and being conducted under common supervision with this EGM to manage cross‐over areas and avoid duplication.

In addition to available and upcoming EGMs, a number of systematic reviews have investigated specific subsets of mental health interventions, outcomes and populations, and will be considered according to our inclusion and exclusion criteria. Barry et al. (2013) reviewed the evidence on the effectiveness of mental health promotion interventions for young people (aged 6–18 years) in school and community settings and Clarke et al. (2015) focused on online prevention interventions. Jordans et al. (2009) synthesised research on interventions addressing child mental health and psychosocial well‐being in conflict settings. More recently, a systematic review assessed the evidence of mental health and psychosocial support programmes for children and adults affected by humanitarian emergencies (Bangpan et al., 2017) and Lloyd‐Reichling et al. (2005) focused on younger children (0–8 years). Bradshaw et al. (2021) reviewed the evidence on scalable school‐based interventions to prevent and address mental health concerns in LMICs. A systematic review identified psychosocial interventions that effectively promote positive mental health and prevent mental health conditions in pregnant and parenting adolescents (Laurenzi et al., 2020). A meta‐analysis identified effective programme components of interventions to promote mental health and prevent mental disorders and risk behaviours during adolescence. In a review of systematic reviews, Das et al. (2016) synthesised the evidence on mental health intervention for adolescents, including but not limited to virtual, individual, group, family and school‐based interventions. Another review investigated programmes aimed at promoting mental health and preventing mental disorders and risk behaviours during adolescence (Skeen et al., 2019). Klasen and Crombag (2013) identified a series of affordable and feasible interventions for children and adolescents in low‐resource settings. van Ginneken et al. (2013) and van Ginneken et al. (2021), respectively, analysed the effectiveness of nonspecialist and primarly‐level worker mental health and psychosocial support interventions on child and adolescent mental health.

We also identified and reviewed relevant intervention guidelines and their supporting evidence to define our scope and identify key linkages. The mhGAP (mental health Gap Action Programme) intervention guidelines first developed by WHO in 2010 and updated in 2016 with most recent evidence, provides guidelines for health providers to address mental, neurological and substance abuse disorders (MNS) in nonspecialist settings (WHO, 2010). Based on a series of reviews, the Helping Adolescents Thrive (HAT) program developed guidelines and toolkits for the promotion of positive mental health and prevention of mental health conditions, self‐harm, substance use and other high‐risk behaviours among adolescents, ages 10‐19 (WHO & UNICEF, 2021). DCP3 (Disease Control Priorities), which lays the ground for global priority MNS, includes a chapter on childhood disorders which identifies maternal mental health and parenting skills interventions as holding key potential to the reduction in prevalence of developmental and mental health conditions (Patel et al., 2016).

#### 1.2.2 Conceptual framework

**Figure 1:**
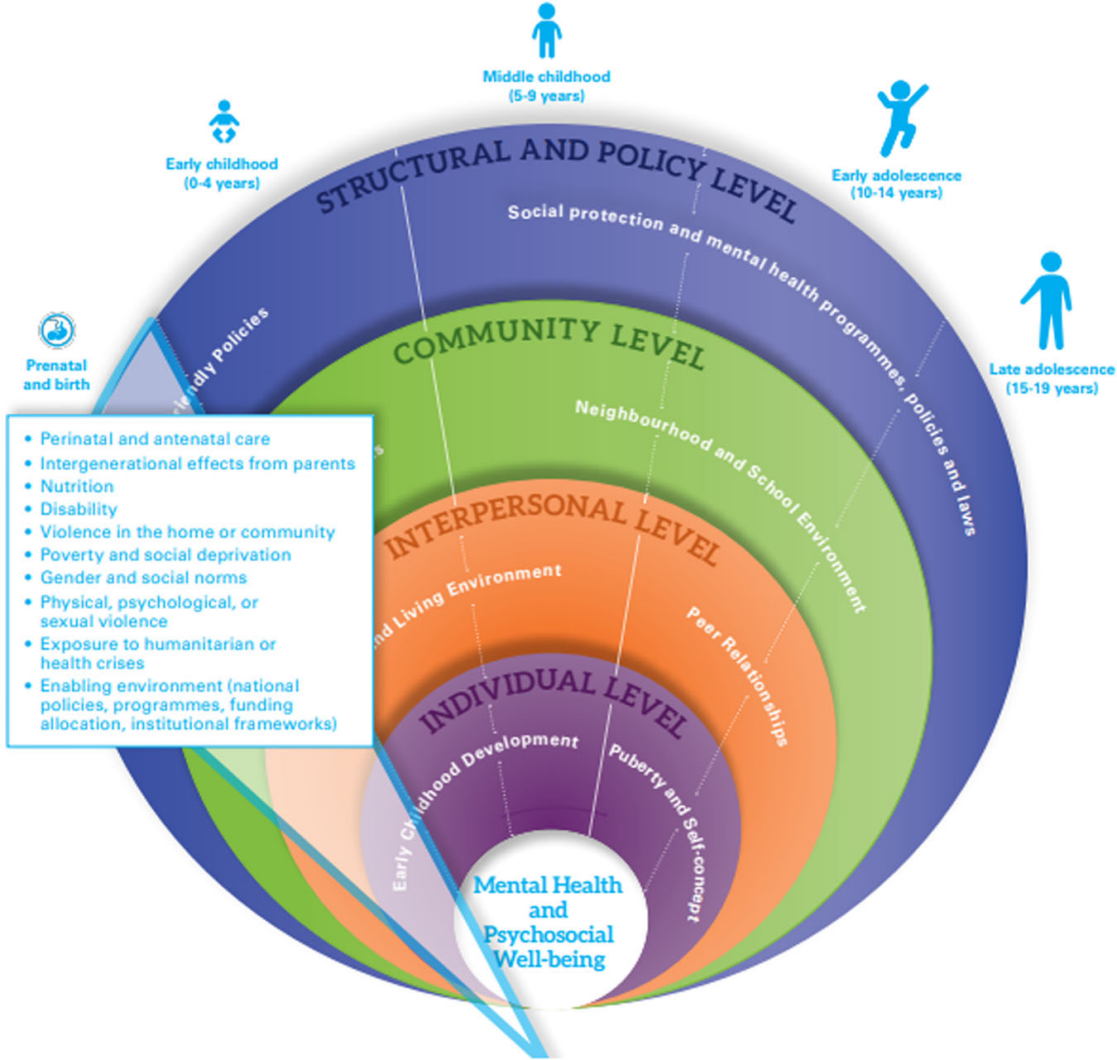
Child and adolescent mental health and psychosocial well‐being: A conceptual framework for research and evidence generation and use (Idele et al., forthcoming)

The conceptual framework guiding this EGM builds upon the mental health research and evidence generation framework proposed by UNICEF Office of Research ‐ Innocenti, which was developed through a series of internal expert consultations to guide evidence and data generation efforts and to support UNICEF's Global Mental Health and Psychosocial Support Framework and programming strategy. The framework recognises that as children and adolescents grow through the life course, their interactions and influences also widen. Their mental health is thus influenced by a myriad of dynamic risk and protective factors at different layers of the social environment across their developmental stages—individual, interpersonal, community and structural and policy levels. Different factors have greater impact at different ages and across these levels (Figure 1).

This framework incorporates elements of other existing frameworks including: (a) the socio‐ecological model (Bronfenbrenner, 1979), which posits that a child's psychosocial well‐being depends on a myriad of factors nested within their broader social environment ranging from the household, through to the community and society levels, and the broader socio‐cultural and policy environment and can be understood as the different delivery platforms by which interventions are deployed; (b) the social determinants of health approach (Marmot & Wilkinson, 2005) which emphasises the role of circumstances in which people are born and grow up, as well as the systems in place to deal with illness; and (c) the life course epidemiology approach (Kuh et al., 2003) which highlights the factors and experiences over the life course and across generations that impact health outcomes at different ages and life stages.

The EGM utilises this framework by organising interventions according to delivery platforms that correspond to the levels in a child's social ecology. Further, the outcomes will be sensitive to the child's life stage and social determinants of health, thereby including child development outcomes as well. Child and adolescent mental health is complex and changes over time according to individual characteristics, relationships, context and experiences. Therefore, child and adolescent mental health is hereby understood to encompass both negative and positive mental health outcomes including well‐being and functioning as well as symptoms of distress or sadness and mental health conditions that may require specialised care.

**Figure 2:**
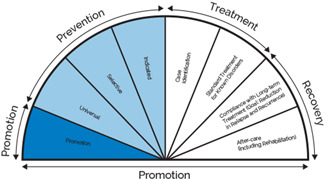
Mental health intervention spectrum (Institute of Medicine, 1994)

Building on this, we will be applying the continuum of care model to categorise mental health interventions as prevention, promotion or treatment, as depicted in Figure 1 (Institute of Medicine, 1994). Mental health promotion interventions aim to enhance well‐being and creative supportive and protective environments for all children and adolescents. Prevention interventions focus on preventing or reducing the risk of developing a mental health condition by targeting modifiable risk factors and can be universal (delivered to the general population, e.g., primary prevention), selective (population sub‐groups deemed to be at risk of mental health conditions developing), or indicated (populations identified at heightened risk for mental health conditions). Treatment interventions are for populations living with a mental health condition. The framework also includes recovery interventions; however, these interventions will be excluded from the EGM (Figure 2).

## 2 OBJECTIVES

The objective of this EGM is to provide an overview of the existing evidence on the effectiveness of interventions aimed at promoting mental health and reducing or preventing mental health conditions among children and adolescents in LMICs.

Consistent with this, the EGM will:

1. Identify, describe and visually represent the existing evidence from systematic reviews and primary studies on the effectiveness of mental health interventions for children and adolescents.
2. Identify existing gaps in evidence to better inform practice and future primary research.
3. Identify clusters of primary studies that offer opportunities for evidence synthesis.

## 3 METHODS

### 3.1 EGM: Definitions

In contrast to systematic reviews, EGMs do not aim to synthesise the outcomes or key messages from available evidence, but instead aim to map the availability of evidence, coverage and gaps across the various dimensions of the EGM framework and make the evidence discoverable, accessible and usable. EGMs provide an overview of the existing evidence on a topic, theme or sector to signpost where evidence exists and/or where it is lacking (Bakrania, 2020).

#### 3.1.1 Types of population

Children and adolescents are defined as any person from 0 to 19 years of age and classified according to UNICEF's age criteria stated as follows: early childhood (0–4 years), middle childhood (5–9 years), early adolescence (10–14 years) and late adolescence (15–19 years). Primary studies where less than 50% of the sample fall within the 0–19 age range or that do not provide sufficient information of age composition will be excluded.

Population subgroups of interest includes children in alternative care, children with disabilities, LGBTQIA + children, ethnic or racial minorities, child workers, married children, street children, children with chronic health conditions, pregnant adolescents and adolescent parents and forcibly displaced children. Studies that disaggregate findings by these population subgroups will be coded as such. We will also note whether studies or reviews focus on girls/females, boys/males and/or other.

LMICs are defined according to the World Bank's regional classification by country gross national income as: low‐income, lower middle‐income, upper middle‐income economies (The World Bank, 2021).

#### 3.1.2 Types of intervention

Mental health and psychosocial support is defined as ‘any type of local or outside support that aims to protect or promote psychosocial well‐being and/or prevent or treat mental disorders’ (Inter‐Agency Standing Committee, 2007, p. 1). As such, the primary aim of mental health and psychosocial support (MHPSS) interventions is to prevent or treat mental health conditions or to promote of mental health and psychosocial well‐being. While there is not agreed upon categorisation of MHPSS interventions, they are hereby organised according to the levels of the social‐ecological model and UNICEF Office of Research—Innocenti's conceptual framework, based on the platform of their delivery:

- Individual and family‐based interventions
- School‐based interventions
- Community‐based interventions
- Digital interventions

The EGM will also include filters for individual, interpersonal and group‐based interventions. Under each of these categories, interventions are further categorised as mental health promotion, prevention of mental health conditions or treatment interventions. This categorisation is based on the continuum of interventions highlighted in the conceptual framework section above, and on the description for these types of interventions provided in the systematic review of MHPSS interventions for conflict‐affected children in LMICs (Jordans et al., 2016).

- Promotion: activities and programs focusing on strengthening positive aspects of mental health and child well‐being.
- Prevention: activities and programs that aim to stop mental health conditions from developing, by acting on the social determinants of mental health that may be known risk factors for certain mental disorders.
- Treatment: activities to reduce symptoms and improve functioning in people with identified mental disorders. We will exclude pharmacological treatment.

The distinction between interventions that enhance mental health and those that lower the risk of or ameliorate mental health conditions is not always clear (Supporting Information Appendix 1; Tol et al., 2015). Therefore, although we include examples of interventions for each intervention type, it should be noted that there may be overlaps, for example, a cognitive behaviour therapy intervention can be classified, depending on its objectives and modules, as promotion if it is focused on building life skills or treatment if it teaches children how to cope with and overcome anxiety). Recovery interventions focused on compliance with long‐term treatment with the goal of reducing relapse and recurrence as well as after care and rehabilitation will be excluded from the EGM. We will exclude studies investigating the effectiveness of neurofeedback.

Table 1 lists the intervention categories and subcategories. The included interventions cover all key mental health and psychosocial support interventions across different contexts and levels of the child's social ecology, organised by platform of delivery (i.e., individual and family, school, community, and digital). The intervention categories included in our EGM are:

**Table 1:** Interventions

Intervention platform	Intervention type	Examples
Individual and family‐based	Prevention	Life‐skills, Maternal and paternal stimulation
	Promotion	Psychoeducation, Parenting education, Exercise
	Treatment	Cognitive behaviour therapy, Interpersonal psychotherapy, Psychological First Aid
School‐based	Prevention	Peer support, Life skills training
	Promotion	School‐based mental health promotion, Peer support, Life skills education
	Treatment	School counselling, Group cognitive behaviour therapy
Community‐based	Prevention	Primary prevention, Child friendly spaces
	Promotion	Stigma reduction, Community‐based mental health advocacy
	Treatment	Task shifting and task sharing interventions, Psychological First Aid in the community
Digital	Prevention	Computerised cognitive behaviour therapy
	Promotion	Online mental health promotion, Online psychoeducation
	Treatment	Online mindfulness‐based CBT

#### 3.1.3 Types of outcomes

The main outcome categories are listed in Table 2. Systematic reviews and primary studies investigating the impact of MHPSS interventions on violence prevention outcomes (addressed in the violence against children EGM; Pundir et al., 2020).

**Table 2:** Outcomes

Outcome domains	Sub domains
*Mental health conditions*	
Internalising conditions	Depression
	Anxiety disorders
	Post‐traumatic stress disorder
	Suicidal behavior
	Eating disorders
Externalising conditions	Oppositional defiant disorder
	Conduct disorder
	Attention deficit hyperactivity disorder
	Alcohol and substance use
Other mental health conditions and symptoms	Other (e.g., hopelessness, anger, risk taking behaviours, cultural symptoms of distress)
*Mental well‐being*	
	Well‐being
	Functioning
	Ability to cope
	Social behaviour
	Social connectedness
	Other well‐being outcomes
*Early childhood development outcomes*	
	Social emotional learning
	Cognitive development
	Executive function
	Emotional regulation
	Behaviour problems

We used the International Classification of Disease (ICD‐11) criteria for including mental health conditions. The terms ‘internalising’ and ‘externalising’ refer to internally and externally focused symptoms of mental health conditions respectively. These well established and widely used groupings are derived from factor analyses of psychological problems identified by clinically referred children and describe behavioural, emotional and social problems, encompass a broad range of mental health issues and are not mutually exclusive. The mental health conditions listed under each of these are based on what emerged as the most relevant categories from our recent rapid review on the impact of child and adolescent mental health outcomes (UNICEF Office of Research—Innocenti, 2021). Further, whilst the medicalization and science around mental health conditions has favoured development of discrete outcomes and measures, positive mental health outcomes are yet to be standardised across the literature, with a high variability across approaches. Although positive mental health is a well‐recognised aspect of mental health, there is no established classification of positive mental health outcomes. In this EGM, we classify positive mental health outcomes according to Inter‐Agency Standing Committee Reference Group for Mental Health and Psychosocial Support in Emergency Settings' Common Monitoring and Evaluation Framework (Inter‐Agency Standing Committee, 2021). In additon, we have also included ‘other’ categories for relevant outcomes that may emerge from the included studies and relate directly to the outcomes described above (e.g., sadness or hopelessness as outcomes related to depression, or self‐efficacy and prosocial behaviours as outcomes related to mental well‐being). To capture key early childhood indicators, we will also be looking at child development outcomes such as social emotional learning and cognitive development. These are critical processes through which children acquire and apply knowledge and skills to cope with challenges, manage interpersonal relationships, manage emotions, solve problems, and make informed decisions. These indicators are also linked to later life mental health outcomes (Black et al., 2017; Patel et al., 2018).

#### 3.1.4 Types of study designs

The study designs which will be included in the EGM are: systematic reviews and effectiveness studies in the form of randomised control trials and quasi‐experimental studies. Mixed‐methods studies with a focus on intervention effectiveness will also be included. These types of study designs match the focus on intervention effectiveness.

We will not include qualitative studies in the EGM or quantitative and mixed‐methods studies with focus on topics beyond intervention effectiveness such as training and capacity building of practitioners and providers, detection and diagnosis of mental health conditions, single case reports of treatments, other EGMs, systematic reviews of reviews, natural experiments and research on mental health policy or legislations.

#### 3.1.5 Types of settings

Interventions delivered in hospital settings such as in‐patient psychiatric care are considered beyond the scope of the map's delivery platforms and will not be included in the EGM.

#### 3.1.6 Publication period and types

Studies published from the year 2010 onwards will be included to consider the most updated and relevant search results. This date coincides with the publication of the World Health Organization's Mental Health Global Action Programme (mhGAP) intervention guidelines, providing recommendations for the treatment of mental health conditions in nonspecialist health settings (WHO, 2010).

Peer‐reviewed reports and academic papers will be included. Protocols of systematic reviews and primary studies will be also included and removed if the review or primary study is identified. Pilots of randomised controlled trials will be excluded. Co‐registered reports will be treated as duplicate reviews with data extracted from the most detailed version. Similarly, if multiple versions of the same systematic review are identified, the latest and most comprehensive version will be considered for inclusion. Commentaries, conceptual or theoretical papers, editorials, conference proceedings and clinical cases will not be included.

#### 3.1.7 Languages

Searches will be conducted in English, but studies and reviews written in any language will be considered for inclusion.

### 3.2 Search methods and sources

#### 3.2.1 Search structure

The search will have four blocks: population, interventions, outcomes, and geography. Where available, we will use the existing search filters (such as EPOC filter for Low‐ and Middle‐Income Countries).

#### 3.2.2 Search process

The information specialist (FS) will design and test the draft search strategy for MEDLINE via Ovid SP (Supporting Information Appendix 2). The strategy will be shared with review team and well as the Advisory Board for commenting and revisions. The final search strategy and search resources will be discussed and agreed within the review team before translation into the syntax of other databases and running the final search. The search will be designed following the Chapter 4 of Cochrane Handbook, peer‐reviewed using PRESS guideline, and reported based on PRISMA‐Search.

#### 3.2.3 Databases

A wide range of bibliographic databases, sources of grey literature, and websites will be searched to cover all the relevant subject areas, geography, and study designs. The primary list of databases is as follows:

1. Systematic reviews
  - Campbell Collaboration
  - 3ie Systematic Review Database
  - Cochrane Library Databases
  - Epistemonikos
  - EPPI Centre Evaluation Database of Education Research
  - PROSPERO
  - Social Systems Evidence
2. Regional sources
  - African Index Medicus
  - African Journals Online
  - Latin American and Caribbean Health Sciences Literature (LILACS)
  - SciELO
3. Social science
  - Applied Social Sciences Index and Abstracts (ASSIA) via ProQuest
  - International Bibliography of Social Sciences (IBSS) via ProQuest
  - Social Policy and Practice via Ovid SP
  - Social Science Citation Index via Web of Science
  - Social Science Database via ProQuest
  - Social Science Research Network (SSRN)
  - Sociological Abstracts (including Social Services Abstracts and Sociological Abstracts) via ProQuest
  - PsycINFO via Ovid SP
4. Web and website searches
  - Bing Site Search (per website, including grey literature)
  - Google Site Search (per website, including grey literature)
  - UN‐affiliated relevant websites
  - Subject‐focused websites
5. Health/medical databases
  - CINAHL via EBSCOhost
  - Embase via Ovid SP
  - ClinicalTrials.gov (Tentative)
  - Global Health via Ovid SP
  - Global Index Medicus
  - MEDLINE via Ovid SP
  - WHO ICTRP (Tentative)
  - WHO's Global Health Library
  - PubMed (excluding MEDLINE)
6. Educational databases
  - Child Development & Adolescent Studies via EBSCOhost
  - Education Resources Information Center (ERIC) via ProQuest
7. Science databases
  - Emerging Sources Citation Index via Web of Science
  - Science Citation Index Expanded via Web of Science
  - Scopus
8. Grey literature
  - Google Scholar (including grey literature)
  - ProQuest Dissertations & Theses Global

##### Screening and study selection

All titles and abstracts, and then full text, will be double screened, with a third‐party arbitrator in the event of disagreement. Screening will be conducted using EPPI‐Reviewer Web (Thomas et al., 2020). The screening tool is given as Supporting Information Appendix 3. Due to time and resource limitations, if the number of retrieved records exceed 10,000 at title and abstract we will employ EPPI‐Reviewer's machine learning and priority screening functions to automatically exclude records with a low probability of meeting the inclusion criteria.

##### Data extraction and management

Data extraction will be conducted using EPPI‐Reviewer Web. Due to the expected large volume of reviews meeting inclusion criteria, a small sample of studies and reviews will be extracted by two reviewers and disagreements will be resolved by consensus. The remaining coding will be conducted by one reviewer independently, in consultation with other reviewers when necessary.

##### Critical appraisal

Due to the expected large volume of studies and reviews meeting our inclusion criteria, we will not be appraising the quality of included studies and reviews. Instead, we will collect data on study design and type of systematic review as well as the number of participants included in each primary study or the number of studies within each review. In the final report, we will discuss the implications and sources of bias introduced by each type of design or review.

##### Methods for mapping

EPPI‐Mapper will be used to develop the EGM.

#### 3.2.4 Analysis and presentation

##### Presentation

Each entry in the map will be a systematic review or a primary study of effectiveness. The final EGM will identify the number of studies covered by the map according to each intervention and outcome dimension. The available evidence will be represented across two dimensions: the rows list interventions and the columns list outcome domains. Each cell will show studies and reviews which contain evidence on that combination of intervention and outcomes or absolute gaps when no evidence exists. The number of included primary studies and reviews will be shown by the size of the bubble and the type of design or review will be indicated by the colour of each bubble. In addition to the dimensions (i.e., interventions and outcomes), Table 3 presents the filters of the EGM.

**Table 3:** Filters for the EGM

Category	Data items
Context	• Publication year
	• Income level: low income; lower‐middle income; upper‐middle income; global (with at least one LMIC study included)
	• Countries
	• Region: East Asia & Pacific, Europe & Central Asia, Latin America & Caribbean, Middle East & North Africa, North America, South Asia, Western Central Africa, Eastern Central Africa
	• Humanitarian context: Study or review explicitly mentions that the MHPSS intervention (as defined by IASC guidelines) was conducted in a humanitarian or conflict‐affected region.
	• COVID‐19: Study or review explicitly mentions having been conducted in the context of the COVID‐19 pandemic or including studies conducted in the context of the COVID‐19 pandemic.
	• Format: Individual, interpersonal and group‐based interventions
Population	• Age group: early childhood (0–4); middle childhood (5–9); early adolescence (10–14); late adolescence (15–19)
	• Gender/sex: girl/female; boy/male; other
	• Sub‐groups: Children in alternative care, children with disabilities, LGBTQIA + children, ethnic or racial minorities, child workers, married children, street children, children with chronic health conditions, pregnant adolescents and adolescent parents and forcibly displaced children.
Study design	• Systematic review—Narrative synthesis
	• Systematic review—Meta‐analysis or meta‐regression
	• Systematic review—Narrative synthesis and meta‐analysis or meta‐regression
	• Primary study—Randomised controlled trial
	• Primary study—Quasi‐experimental study
	• Primary study—Mixed‐methods study
	• Protocol of systematic review or RCT
	• Threshold of number of participants or number of studies

##### Planned analysis

The EGM report will provide tabulations and/or graphs of the number of studies, with accompanying narrative description, by:

- Intervention category and subcategory
- Outcome domain and subdomain
- Region and country
- Year
- Study type
- Type of intervention
- Population sub‐groups

#### 3.2.5 Stakeholder engagement

This EGM is guided by the feedback and input of an Advisory Group composed of experts, researchers, practitioners and advocates from the field of global child and adolescent mental health. Age, gender, field of expertise and geographic focus have been considered when inviting members to join the Advisory Group. The group will be engaged at all stages of the EGM process to review and comment on the EGM protocol, EGM online tool, identify ongoing primary studies and systematic reviews and final report and to provide advice on dissemination channels.

## CONTRIBUTIONS OF AUTHORS

### 1 Roles and responsibilities

Manasi Sharma, PhD (MS) is a global mental health researcher, with expertise in child and adolescent health, psychiatric epidemiology, and implementation science. Her research focuses on mixed methods approaches to the development and evaluation of mental health interventions in low‐ and middle‐income countries. Camila Perera, PhD (CP) is a global mental health researcher with experience in conducting systematic reviews. Her main research interest and recent work focus on adverse childhood experiences and cultural adaptations of psychological interventions. Alessandra Ipince (AI) is a social anthropologist with experience in youth‐led participatory research and systematic review methodologies, including EGMs. Shivit Bakrania (SB) is the Knowledge Management Specialist at UNICEF's Office of Research and has experience as the Principal Investigator of high‐profile evidence and gap map and evidence synthesis projects for United Kingdom's Foreign, Commonwealth and Development Office and UNICEF. Farhad Shokraneh, PhD (FS) is an Information Specialist and Assistant Professor at the University of Nottingham. He is a co‐author of the Cochrane Handbook for Systematic Reviews of Interventions, has conducted multiple systematic reviews and developed a new methodology to improve searching to identify several interventions from developing countries not covered by traditional search strategies. Priscilla Idele, PhD (PI) is the Deputy Director at the UNICEF Office of Research in Florence, Italy. Her work over the past 30 years has focused on strategic research direction and leadership and she has broad experience in international development, child rights, protection and public health issues. David Anthony (DA) has extensive experience in managerial and strategy development, partnerships and engagement at setting policy agendas and in translating them into practical action and results and provides strategic guidance to this EGM. Prerna Banati, PhD (PB) is a senior advisor specialising in global and regional policy and program issues, as they relate to adolescents and young people. She has close to 20 years of international development experience with the UN and other international organisations, providing strategic advice on adolescent development and sensitive issues.

- Content: MS, CP
- EGM methods: SB, AI, CP
- Information retrieval: FS
- Guidance: PI, DA, PB

## DECLARATIONS OF INTEREST

None known.

## PLANS FOR UPDATING THE EGM

Once completed, the EGM will be updated yearly, depending on the need of an update (availability of new reviews and primary studies). Regular updates are also subject to availability of funding. If funding is available, UNICEF Office of Research—Innocenti takes responsibility for updating the review.

## SOURCES OF SUPPORT

**Internal sources**

- UNICEF Office of Research—Innocenti, Italy
  The funding for this EGM is provided by UNICEF Office of Research—Innocenti.

**External sources**

- Not Applicable

## Supporting information

Supporting information.
