# Supplementary material for: PROTOCOL: Child and adolescent mental health and psychosocial support interventions: An evidence and gap map of low‐ and middle‐income countries
Source: Campbell Syst Rev. 2022 Feb 16;18(1):e1221. doi: 10.1002/cl2.1221 (PMC8848633; doi:10.1002/cl2.1221)
Supplement: Supplementary file 1 — Supporting information. [file CL2-18-e1221-s001.docx]

# Appendices

## 1 Definitions

This protocol reflects the terminology that has been used for the State of the World’s Children (SOWC) 2021 report on mental health ([UNICEF 2021](#REF-UNICEF-2021)). As described in the SOWC 2021, the term ‘mental health condition’ describes a wide range of conditions that can vary in severity from mild and temporary to severe and lifelong. The World Health Organization has also used mental health condition in some materials to indicate “mental, neurological and substance use disorders, suicide risk and associated psychosocial, cognitive and intellectual disabilities.” However, the field of mental health uses multiple terms, some of which have more specific meanings. The terms used for negative mental health outcomes collectively include mental disorder, mental ill-health, mental health condition and psychosocial disability. We will be using the term mental health conditions in this EGM. We will also be using the terms mental health and psychosocial well-being to refer to positive mental health outcomes.

## 2 Search strategy for MEDLINE via Ovid SP

*Block 1: Geography*

1. (Afghanistan OR Albania OR Algeria OR American Samoa OR Angola OR "Antigua And Barbuda" OR Antigua OR Barbuda OR Argentina OR Armenia OR Armenian OR Aruba OR Azerbaijan OR Bahrain OR Bangladesh OR Barbados OR Republic Of Belarus OR Belarus OR Byelarus OR Belorussia OR Byelorussian OR Belize OR British Honduras OR Benin OR Dahomey OR Bhutan OR Bolivia OR "Bosnia And Herzegovina" OR Bosnia OR Herzegovina OR Botswana OR Bechuanaland OR Brazil OR Brasil OR Bulgaria OR Burkina Faso OR Burkina Fasso OR Upper Volta OR Burundi OR Urundi OR Cabo Verde OR Cape Verde OR Cambodia OR Kampuchea OR Khmer Republic OR Cameroon OR Cameron OR Cameroun OR Central African Republic OR Ubangi Shari OR Chad OR Chile OR China OR Colombia OR Comoros OR Comoro Islands OR Iles Comores OR Mayotte OR Democratic Republic Of The Congo OR Democratic Republic Congo OR Congo OR Zaire OR Costa Rica OR "Cote D’ivoire" OR "Cote D’ Ivoire" OR Cote Divoire OR Cote D Ivoire OR Ivory Coast OR Croatia OR Cuba OR Cyprus OR Czech Republic OR Czechoslovakia OR Djibouti OR French Somaliland OR Dominica OR Dominican Republic OR Ecuador OR Egypt OR United Arab Republic OR El Salvador OR Equatorial Guinea OR Spanish Guinea OR Eritrea OR Estonia OR Eswatini OR Swaziland OR Ethiopia OR Fiji OR Gabon OR Gabonese Republic OR Gambia OR "Georgia (Republic)" OR Georgian OR Ghana OR Gold Coast OR Gibraltar OR Greece OR Grenada OR Guam OR Guatemala OR Guinea OR Guinea Bissau OR Guyana OR British Guiana OR Haiti OR Hispaniola OR Honduras OR Hungary OR India OR Indonesia OR Timor OR Iran OR Iraq OR Isle Of Man OR Jamaica OR Jordan OR Kazakhstan OR Kazakh OR Kenya OR "Democratic People’s Republic Of Korea" OR Republic Of Korea OR North Korea OR South Korea OR Korea OR Kosovo OR Kyrgyzstan OR Kirghizia OR Kirgizstan OR Kyrgyz Republic OR Kirghiz OR Laos OR Lao Pdr OR "Lao People's Democratic Republic" OR Latvia OR Lebanon OR Lebanese Republic OR Lesotho OR Basutoland OR Liberia OR Libya OR Libyan Arab Jamahiriya OR Lithuania OR Macau OR Macao OR Republic Of North Macedonia OR Macedonia OR Madagascar OR Malagasy Republic OR Malawi OR Nyasaland OR Malaysia OR Malay Federation OR Malaya Federation OR Maldives OR Indian Ocean Islands OR Indian Ocean OR Mali OR Malta OR Micronesia OR Federated States Of Micronesia OR Kiribati OR Marshall Islands OR Nauru OR Northern Mariana Islands OR Palau OR Tuvalu OR Mauritania OR Mauritius OR Mexico OR Moldova OR Moldovian OR Mongolia OR Montenegro OR Morocco OR Ifni OR Mozambique OR Portuguese East Africa OR Myanmar OR Burma OR Namibia OR Nepal OR Netherlands Antilles OR Nicaragua OR Niger OR Nigeria OR Oman OR Muscat OR Pakistan OR Panama OR Papua New Guinea OR New Guinea OR Paraguay OR Peru OR Philippines OR Philipines OR Phillipines OR Phillippines OR Poland OR "Polish People's Republic" OR Portugal OR Portuguese Republic OR Puerto Rico OR Romania OR Russia OR Russian Federation OR Ussr OR Soviet Union OR Union Of Soviet Socialist Republics OR Rwanda OR Ruanda OR Samoa OR Pacific Islands OR Polynesia OR Samoan Islands OR Navigator Island OR Navigator Islands OR "Sao Tome And Principe" OR Saudi Arabia OR Senegal OR Serbia OR Seychelles OR Sierra Leone OR Slovakia OR Slovak Republic OR Slovenia OR Melanesia OR Solomon Island OR Solomon Islands OR Norfolk Island OR Norfolk Islands OR Somalia OR South Africa OR South Sudan OR Sri Lanka OR Ceylon OR "Saint Kitts And Nevis" OR "St. Kitts And Nevis" OR Saint Lucia OR "St. Lucia" OR "Saint Vincent And The Grenadines" OR Saint Vincent OR "St. Vincent" OR Grenadines OR Sudan OR Suriname OR Surinam OR Dutch Guiana OR Netherlands Guiana OR Syria OR Syrian Arab Republic OR Tajikistan OR Tadjikistan OR Tadzhikistan OR Tadzhik OR Tanzania OR Tanganyika OR Thailand OR Siam OR Timor Leste OR East Timor OR Togo OR Togolese Republic OR Tonga OR "Trinidad And Tobago" OR Trinidad OR Tobago OR Tunisia OR Turkey OR Turkmenistan OR Turkmen OR Uganda OR Ukraine OR Uruguay OR Uzbekistan OR Uzbek OR Vanuatu OR New Hebrides OR Venezuela OR Vietnam OR Viet Nam OR Middle East OR West Bank OR Gaza OR Palestine OR Yemen OR Yugoslavia OR Zambia OR Zimbabwe OR Northern Rhodesia OR Global South OR Africa South Of The Sahara OR Sub-Saharan Africa OR Subsaharan Africa OR Africa, Central OR Central Africa OR Africa, Northern OR North Africa OR Northern Africa OR Magreb OR Maghrib OR Sahara OR Africa, Southern OR Southern Africa OR Africa, Eastern OR East Africa OR Eastern Africa OR Africa, Western OR West Africa OR Western Africa OR West Indies OR Indian Ocean Islands OR Caribbean OR Central America OR Latin America OR "South And Central America" OR South America OR Asia, Central OR Central Asia OR Asia, Northern OR North Asia OR Northern Asia OR Asia, Southeastern OR Southeastern Asia OR South Eastern Asia OR Southeast Asia OR South East Asia OR Asia, Western OR Western Asia OR Europe, Eastern OR East Europe OR Eastern Europe OR Developing Country OR Developing Countries OR Developing Nation? OR Developing Population? OR Developing World OR Less Developed Countr* OR Less Developed Nation? OR Less Developed Population? OR Less Developed World OR Lesser Developed Countr* OR Lesser Developed Nation? OR Lesser Developed Population? OR Lesser Developed World OR Under Developed Countr* OR Under Developed Nation? OR Under Developed Population? OR Under Developed World OR Underdeveloped Countr* OR Underdeveloped Nation? OR Underdeveloped Population? OR Underdeveloped World OR Middle Income Countr* OR Middle Income Nation? OR Middle Income Population? OR Low Income Countr* OR Low Income Nation? OR Low Income Population? OR Lower Income Countr* OR Lower Income Nation? OR Lower Income Population? OR Underserved Countr* OR Underserved Nation? OR Underserved Population? OR Underserved World OR Under Served Countr* OR Under Served Nation? OR Under Served Population? OR Under Served World OR Deprived Countr* OR Deprived Nation? OR Deprived Population? OR Deprived World OR Poor Countr* OR Poor Nation? OR Poor Population? OR Poor World OR Poorer Countr* OR Poorer Nation? OR Poorer Population? OR Poorer World OR Developing Econom* OR Less Developed Econom* OR Lesser Developed Econom* OR Under Developed Econom* OR Underdeveloped Econom* OR Middle Income Econom* OR Low Income Econom* OR Lower Income Econom* OR Low Gdp OR Low Gnp OR Low Gross Domestic OR Low Gross National OR Lower Gdp OR Lower Gnp OR Lower Gross Domestic OR Lower Gross National OR Lmic OR Lmics OR Third World OR Lami Countr* OR Transitional Countr* OR Emerging Economies OR Emerging Nation? Or Afghan OR Afghans OR Afghani OR Albanian? Algerian? OR American Samoan? OR Angolan? OR Antiguan? OR Barbudan? OR Argentine? OR Argentinian? OR Argentinean? OR Armenian? OR Aruban? OR Azerbaijani? OR Bahraini? OR Bangladeshi? OR Bangalees OR Bajan? OR Belarusian? OR Byelorussian? OR Belizean? OR Beninese? OR Bhutanese OR Bolivian? OR Bosnian? OR Botswana OR Batswana OR Brazilian? OR Brasilian? OR Bulgarian? OR Burkinabe OR Burkinese OR Burundian? OR Cape Verdean? OR Cabo Verdean? OR Cambodian? OR Khmer OR Cameroonian? OR Central African? OR Chadian? OR Chilean? OR Chinese OR Colombian? OR Comorian? OR Congolese OR Costa Rican? OR Ivorian? OR Croatian? OR Cuban? OR Cypriot? OR Czech? OR Djiboutian? OR Dominican? OR Ecuadorian? OR Egyptian? OR Salvadoran? OR Equatorial Guinean? OR Equatoguinean? OR Eritrean? OR Estonian? OR Swazi? OR Swati? OR Ethiopian? OR Fijian OR Gabonese OR Gabonaise OR Gambian? OR Georgian? OR Ghanaian? OR Gibraltarian? OR Greek? OR Grenadian? OR Guamanian? OR Guatemalan? OR Guinean? OR Bissau Guinean? OR Guyanese OR Haitian? OR Honduran? OR Hungarian? OR Indian? OR Indonesian? OR Iranian? OR Iraqian? OR Iraqi? OR Manx OR Jamaican? OR Jordanian? OR Kazakhstani? OR Kenyan? OR Kirabati OR Kirabatian? OR North Korean? OR Korean? OR Kosovar? OR Kosovan? OR Kyrgyz* OR Lao OR Laotian? OR Latvian? OR Lebanese OR Lesothan? OR Lesothonian? OR Mosotho OR Basotho OR Liberian? OR Libyan? OR Lithuanian? OR Macanese OR Macedonian? OR Malagasy OR Madagascan? OR Malawian? OR Malaysian? OR Maldivian? OR Malian? OR Maltese OR Marshallese? OR Mauritanian? OR Mauritian? OR Mexican? OR Micronesian? OR Moldovan? OR Mongolian? OR Mongol OR Montenegrin? OR Moroccan? OR Mozambican? OR Burmese OR Myanma OR Namibian? OR Nauruan? OR Nepali OR Nepalese OR Netherlands Antillean? OR Nicaraguan? OR Nigerien? OR Nigerian? OR Northern Mariana Islander? OR Mariana? OR Omani? OR Pakistani? OR Palauan? OR Panamanian? OR Papua New Guinean? OR Paraguayan? OR Peruvian? OR Philippine? OR Philipine? OR Phillipine? OR Phillippine? OR Filipino? OR Filipina? OR Polish OR Pole OR Poles OR Portuguese OR Puerto Rican? OR Romanian? OR Russian? OR Soviet People OR Soviet Population OR Rwandan? OR Rwandese OR Ruandan? OR Ruandese OR Samoan? OR Sao Tomean? OR Santomean? OR Saudi Arabian? OR Saudi? OR Senegalese OR Serbian? OR Montenegrin? OR Seychellois OR Seychelloise? OR Sierra Leonean? OR Slovak? OR Slovene? OR Solomon Islander? OR Somali? OR South African? OR South Sudanese OR Sri Lankan? OR Ceylonese OR Kittitian? OR Nevisian? OR Saint Lucian? OR Vincentian? OR Sudanese OR Surinamese? OR Syrian? OR Tajik? OR Tajikistani? OR Tanzanian? OR Tanganyikan? OR Thai OR Timorese? OR Togolese OR Tongan? OR Trinidadian? OR Tobagonian? OR Tunisian? OR Turk? OR Turkish OR Turkmen? OR Tuvaluan? OR Ugandan? OR Ukrainian? OR Uruguayan? OR Uzbek? OR Vanuatu* OR Venezuelan? OR Vietnamese OR Yemeni? OR Yemenite? OR Yemenese OR Yugoslav? OR Yugoslavian? OR Zambian? OR Zimbabwean? OR Taiwan* OR Palestin* OR Global* OR World* OR International* OR WHO OR UN OR Nations  Universal* OR Humanitarian? OR Refug* OR "Low and Middle Income" OR "Low Income" OR "Middle Income" OR Foster Care).ti,ab,sh,kf. OR (War OR Conflict).ti.

*Block 2: Population*

2. exp Adolescent/ OR exp Child/ OR exp Infant/ OR Young Adult/ OR (Child OR Children OR Childhood OR Kids OR Adolescen* OR Teen OR Teens OR Teenager OR Teenagers OR Youth OR Youths OR Young OR Juvenile OR Boys OR Girls OR Preschool* OR Highschool* OR School* OR Classroom* OR Class Room* OR College OR Universit* OR Pupil* OR Student* OR Parent* OR Guardian* OR Mother* OR Father* OR Matern* OR Patern* OR Infants OR Newborn* OR Babies OR Toddlers).ti.

*Block 3: Outcomes*

3. Mental Health/ OR Anxiety/ OR Anxiety Disorders/ OR Depression/ OR Depressive Disorder/ OR "Feeding and Eating Disorders"/ OR "Attention Deficit Disorder with Hyperactivity"/ OR Conduct Disorder/ OR exp Sleep Wake Disorders/ OR exp Stress Disorders, Traumatic/ OR Stress Disorders, Post-Traumatic/ OR exp Somatoform Disorders/ OR exp Substance-Related Disorders/ OR exp Self-Injurious Behavior/ OR (Emotion* OR Mental OR Psych* OR Well?being).ti. OR (Anx* OR Eating Disorder? OR Anorexia Nervosa OR Avoidant Restrictive Food Intake Disorder? OR Binge-Eating Disorder? OR Bulimia Nervosa OR Diabulimia OR Food Addiction OR Night Eating Syndrome OR Depress* OR Attention Deficit OR Hyperactiv* OR ADHD OR Conduct Disorder* OR Sleep Disorder? OR Wake Disorder? OR Dyssomnia? OR Parasomnia? OR Insomnia OR Sleep Deprivation OR Somatoform Disorder? OR Body Dysmorphic Disorder* OR Body Integrity Identity Disorder* OR Conversion Disorder* OR Globus Sensation OR Factitious Disorder* OR Munchausen Syndrome OR Hypochondriasis OR Neurasthenia OR ((Substance OR Alcohol OR Amphetamine OR Cocaine OR Inhalant OR Marijuana OR Opium OR Phencyclidine OR Tobacco) adj1 (Use* OR Abuse*)) OR Narcotic* OR Trauma* OR Posttrauma* OR Suicid* OR Self-Harm OR Self-Injur* OR Self-Destruct* OR Self Mutilat* OR PTSD OR Mental Health OR Mental Hygiene OR Mental Well?being OR Subjective Well?being OR Psychological Well?Being OR Oppositional Defiant Disorder? OR Emotional Disorder? OR Internali* OR Externali* OR Cognitive Development OR Socioemotional Learning OR Life Satisfaction OR Resilience OR Coping OR Functioning OR Prosocial Behavio?r*).ti,ab.

*Block 4: Interventions*

4. Psychotherapy/ OR Art Therapy/ OR exp Behavior Therapy/ OR Emotion-Focused Therapy/ OR Interpersonal Psychotherapy/ OR Psychosocial Intervention/ OR exp Socioenvironmental Therapy/ OR exp Cognitive Behavioral Therapy/ OR Internet-Based Intervention/ OR Primary Prevention/ OR exp Social Support/ OR Adaptation, Psychological/ OR Assertiveness/ OR Yoga/ OR Exercise/ OR Counseling/ OR (Interven* OR Trial* OR Promot* OR Prevent* OR Manag* OR Treat* OR Reduc* OR Improv* OR Therap* OR Impact* OR Effect* OR Affect* OR Evaluat* OR Influenc* OR Program* OR Implement* OR Plan* OR Policy OR Policies OR Efficacy OR Training OR Support* OR Care OR Healthcare).ti. OR (CBT OR Behavio* Therap* OR Behavio* Intervention* OR Cognit* Therap* OR Cognit* Intervention* OR Cognitive Behavio* OR Parenting OR Parental Intervention* OR Parental Education OR Parental Training OR Mental Health Literacy OR Psychoeducation* OR Psycho-Education* OR Assertiveness OR Trauma Healing OR Graded Exposure OR Yoga OR Exercise OR Movement Therapy OR Physical Activit* OR Communication Skill* OR Life Skill* OR Living Skill* OR Living Well OR Mindfulness OR Eye Movement Desensitization Reprocessing OR Eye Movement Desensitisation Reprocessing OR EMDR OR Art OR Arts OR Psychological First Aid* OR Mental Health First Aid* OR MHFA OR Peer Support OR Counsel* OR Family Strengthening OR Stigma Reduction OR Anti-Stigma* OR Antistigma* OR Psychotherap* OR Stepped Care OR Collaborative Care OR Psychosocial Support* OR Mental Health Treatment OR Primary Prevention OR Interpersonal Therapy OR Wellness Intervention* OR Positive Psychology OR Mental Health Advocacy OR MHPSS  OR Mental Health Care OR Mental Healthcare OR Family Intervention* OR Family Therap* OR Mental Health Promotion OR Mental Hygiene OR Mental Health Support OR Psychological Support OR Task Shifting OR Task Sharing OR Maternal Stimulation OR Paternal Stimulation OR Child Friendly OR Community Outreach OR Social Prescri*).ti,ab.

***Final Lines***

5. AND/1-4

6. Limit 5 to YR="2010 -Current"

## 3 Screening tool

| **General screening questions** | **No** | **Yes** | **Unclear** |
| --- | --- | --- | --- |
| • Make quick judgements to exclude documents based on the following screening questions.  • If you cannot exclude, or if you’re unclear after checking against all criteria, then it must be forwarded for abstract screening. | | | |
| **Was the study/review published before 2010?** |  |  |  |
| If yes, then exclude.  *If the study is a key systematic review in the field however, it can be saved in a separate folder for the background section.* | | | |
| **Does the study/review include children or adolescents (ages 0-19)?** |  |  |  |
| If no, then exclude.  *There may be some studies that include older age groups as well; as long as it includes anyone aged 0-19 years, it should be included. We will deal with the disaggregation of data for younger ages at the next stage.* | | | |
| **Is the intervention a biomedical trial of a product, medication or procedure?** |  |  |  |
| If yes, then exclude. | | | |
| **Does the study/review include focus on or include Low- and Middle-Income Countries (LMICs)?** |  |  |  |
| If no, then exclude.  *For systematic reviews, there should be at least one primary study from an LMIC included.* | | | |
| **Does the study/review focus on interventions for mental health promotion, prevention or treatment?** |  |  |  |
| If no, then exclude. | | | |
| **Does the article include any of the following study designs?**  **Randomized controlled trials, quasi‐experimental studies, mixed-methods studies, systematic reviews or meta-analyses.** |  |  |  |
| If no, then exclude. | | | |
